# Genome-wide analysis of differentially expressed profiles of mRNAs, lncRNAs and circRNAs during *Cryptosporidium baileyi* infection

**DOI:** 10.1186/s12864-018-4754-2

**Published:** 2018-05-10

**Authors:** Guan-Jing Ren, Xian-Cheng Fan, Ting-Li Liu, Sha-Sha Wang, Guang-Hui Zhao

**Affiliations:** Department of Parasitology, College of Veterinary Medicine, Northwest A&F University, Yangling, 712100 China

**Keywords:** Differentially expressed profiles, mRNAs, lncRNAs, circRNAs, *Cryptosporidium baileyi*

## Abstract

**Background:**

*Cryptosporidium baileyi* is the most common *Cryptosporidium* species in birds. However, effective prevention measures and treatment for *C. baileyi* infection were still not available. Long non-coding RNAs (lncRNAs) and circular RNAs (circRNAs) play important roles in regulating occurrence and progression of many diseases and are identified as effective biomarkers for diagnosis and prognosis of several diseases. In the present study, the expression profiles of host mRNAs, lncRNAs and circRNAs associated with *C. baileyi* infection were investigated for the first time.

**Results:**

The tracheal tissues of experimental (*C. baileyi* infection) and control chickens were collected for deep RNA sequencing, and 545,479,934 clean reads were obtained. Of them, 1376 novel lncRNAs were identified, including 1161 long intergenic non-coding RNAs (lincRNAs) and 215 anti-sense lncRNAs. A total of 124 lncRNAs were found to be significantly differentially expressed between the experimental and control groups. Additionally, 14,698 mRNAs and 9085 circRNAs were identified, and significantly different expressions were observed for 1317 mRNAs and 104 circRNAs between two groups. Bioinformatic analyses of gene ontology (GO) and Kyoto Encyclopedia of Genes and Genomes (KEGG) pathway for their targets and source genes suggested that these dysregulated genes may be involved in the interaction between the host and *C. baileyi*.

**Conclusions:**

The present study revealed the expression profiles of mRNAs, lncRNAs and circRNAs during *C. baileyi* infection for the first time, and sheds lights on the roles of lncRNAs and circRNAs underlying the pathogenesis of *Cryptosporidium* infection.

**Electronic supplementary material:**

The online version of this article (10.1186/s12864-018-4754-2) contains supplementary material, which is available to authorized users.

## Background

Cryptosporidiosis is one of the most prevalent parasitic diseases in domestic and wild birds, and it has been reported in more than 30 avian species worldwide [1, 2]. Currently, four species of avian *Cryptosporidium*, namely *Cryptosporidium meleagridis* [3], *Cryptosporidium baileyi* [4], *Cryptosporidium galli* [5] and *Cryptosporidium avium* (previous avian genotype V) [6], and 12 genotypes (black duck genotype, Eurasian woodcock genotype, avian genotypes I–IV, VI and goose genotypes I–V) have been recognized [7, 8]. Among them, *C. baileyi*, the most common *Cryptosporidium* species in birds, has been reported in a wide range of avian hosts worldwide, especially in chickens [9, 10]. Infection of *C. baileyi* causes respiratory diseases, presenting symptoms of dyspnoea, coughing, rales and sneezing [4, 10, 11], and sometimes is associated with high morbidity and mortality, especially in broiler chickens [10], leading to considerable economic losses to the poultry industry [12–14]. In addition, *C. baileyi* has been observed in the stool of an immunodeficient man, suggesting the zoonotic potential of this species [15]. However, no effective prevention measures or treatments against *C. baileyi* infection have been developed [16].

In the process of attachment, internalization, and formation of parasitophorous vacuoles [17–20], *Cryptosporidium* molecules (eg. rhoptry protein, microneme protein, dense granules) were released into host cells [21, 22] and the transcriptome of host was dysregulated [23]. Seventy-three genes were upregulated and 74 were downregulated in human HCT-8 cells infected with *C. parvum* [24], and these differentially expressed genes are associated with cell communication, signal transduction and amino acid metabolic processes. Previous studies have indicated that non-coding RNA (ncRNA) molecules were also involved in host-*Cryptosporidium* interaction process, and aberrant expressions of microRNAs (miRNAs) were detected in *C. parvum* infecting epithelial cells [25, 26].

Recently, two new classes of ncRNA, namely long non-coding RNAs (lncRNAs) and circular RNAs (circRNAs), were discovered [27, 28], and they were found to play an important role in regulating occurrence and progression of many diseases [29–31]. LncRNAs are a group of non-coding RNAs longer than 200 nt [32] and dysregulated lncRNAs play important roles in progression of cells proliferation, invasiveness and metastasis of breast cancer [33], lung cancer [34] and colorectal cancer [35] through *cis*- and *trans*-regulation of gene expressions [36, 37] and miRNA sponges [38, 39]. In the abnormal activity of biological processes, such as cell proliferation, cell motility, immune, or inflammation response from diseases like cardiovascular disorders, inflammatory and autoimmune disease, lncRNAs also participate in the disease processes and contribute to controlling the gene regulatory network of host-pathogen interactions [40–42]. On the other hand, the function of circRNAs remains largely unknown. Previous studies have showed that it could harbor specific miRNAs as miRNA sponges [43–45] to suppresses miRNA activity, resulting in increased levels of miRNA targets, influence the expression of cytokines [46], promote cell cycles and inhibit cell apoptosis to act as a candidate oncogene [47, 48]. Additionally, both lncRNAs and circRNAs have been identified as effective biomarkers for diagnosis and prognosis of diseases [49, 50]. However, the genome-wide expression and functional roles of lncRNAs and circRNAs in *Cryptosporidium* infection are unclear. Here, we investigated the expression profiles of mRNAs, lncRNAs and circRNAs associated with *C. baileyi* infection for the first time. Our findings would provide baseline data for developing novel diagnostic and therapeutic targets for avian cryptosporidiosis.

## Results

### Identification of lncRNAs in chicken tracheal tissue

**Fig. 1:**
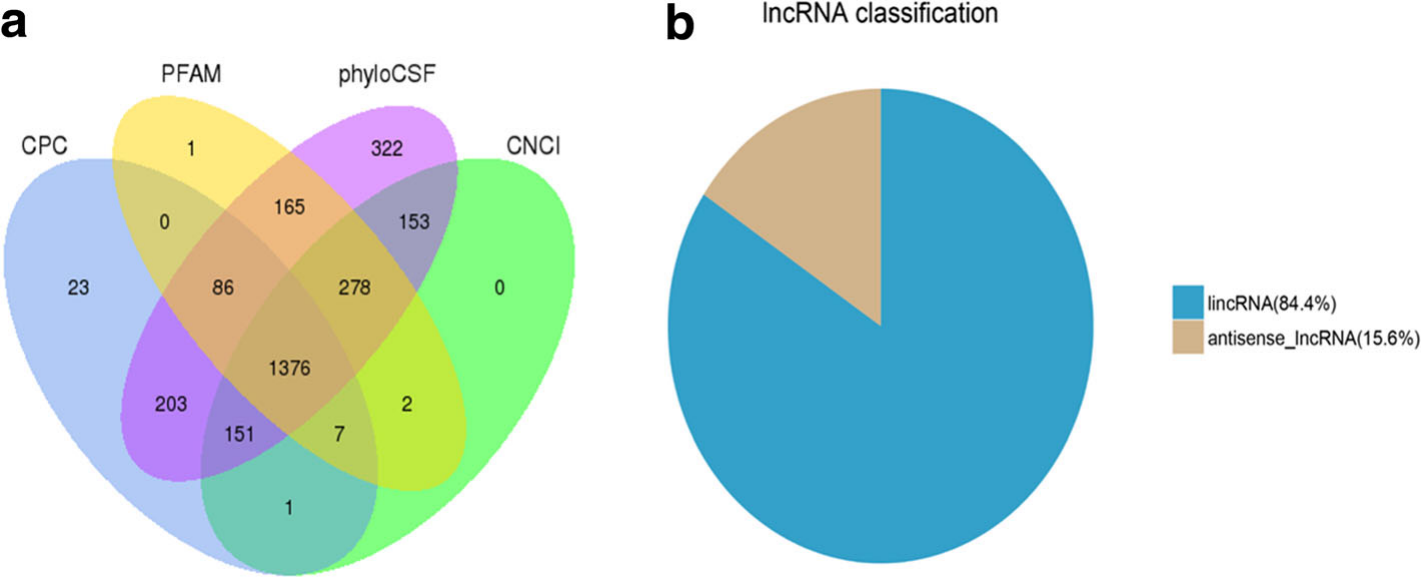
Screening and classification of the candidate lncRNAs in chicken trachea transcriptome. **a** Venn diagrams of coding potential analysis according to strict criteria. Four tools (CPC, CNCI, PFAM, and PhyloCSF) were used to analyze the coding potential of lncRNAs. Those simultaneously shared by four analytical tools were designated as candidate lncRNAs and used in subsequent analyses. **b** Classification of the two subtypes of lncRNAs

A total of 559,574,746 raw reads were produced from the Illumina HiSeq 4000 platform. After abandoned adaptor sequences and low-quality sequences, 545,479,934 clean reads (accounting for 81.82 Gb) were obtained, and the percentage of clean reads among raw data in each library ranged from 96.30 to 98.20%. Subsequently, the clean reads were mapped to the latest *Gallus gallus* reference genome. Firstly, considering the characteristics of lncRNA sequences (≥ 200 nt, exon count ≥2) and their differences from other classes of RNAs (eg. mRNAs, rRNAs, tRNAs, snRNAs, snoRNAs, pre-miRNAs, and pseudogenes), the transcripts were classified into different subtypes using both Scripture beta2 and Cufflinks (v2.1.1). 93.98% of the identified transcripts (50,857) were known as the reference transcripts, whereas 6.02% (3061) were the presumed lncRNAs. To further confirm these 3061 lncRNAs, 2823 transcripts were obtained after filter of the low expression transcripts with FPKM < 0.5. Finally, coding potential analysis was performed using the software CNCI, CPC, Pfam-scan, and PhyloCSF. After being screened by rigorous criteria and four analytic tools, a total of 1376 lncRNAs from the tracheal tissue of chickens were identified and subjected to further analysis (Fig. 1a). The 1376 lncRNAs were composed of 1161 (84.4%) long intergenic non-coding RNAs (lincRNAs) and 215 (15.6%) anti-sense lncRNAs (Additional file 1), nevertheless, other types of lncRNAs such as intronic lncRNAs, sense lncRNAs and bidirectional lncRNAs were not detected in our study (Fig. 1b).

### Differentially expressed profiles of mRNAs, lncRNAs and circRNAs by RNA sequencing

**Fig. 2:**
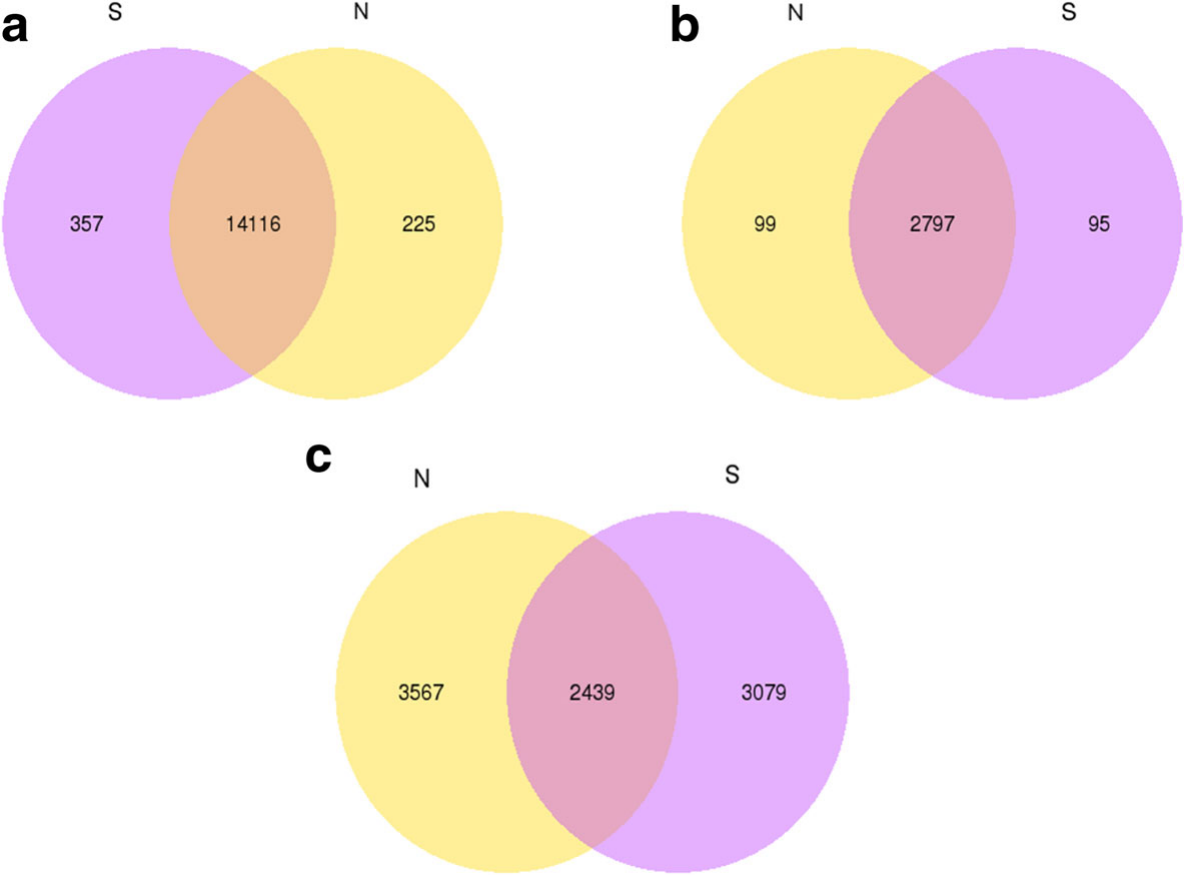
Venn diagram displaying the number of overlap mRNAs (**a**), lncRNAs (**b**) and circRNAs (**c**) in the experimental group (S group) compared with the control group (N group)

**Fig. 3:**
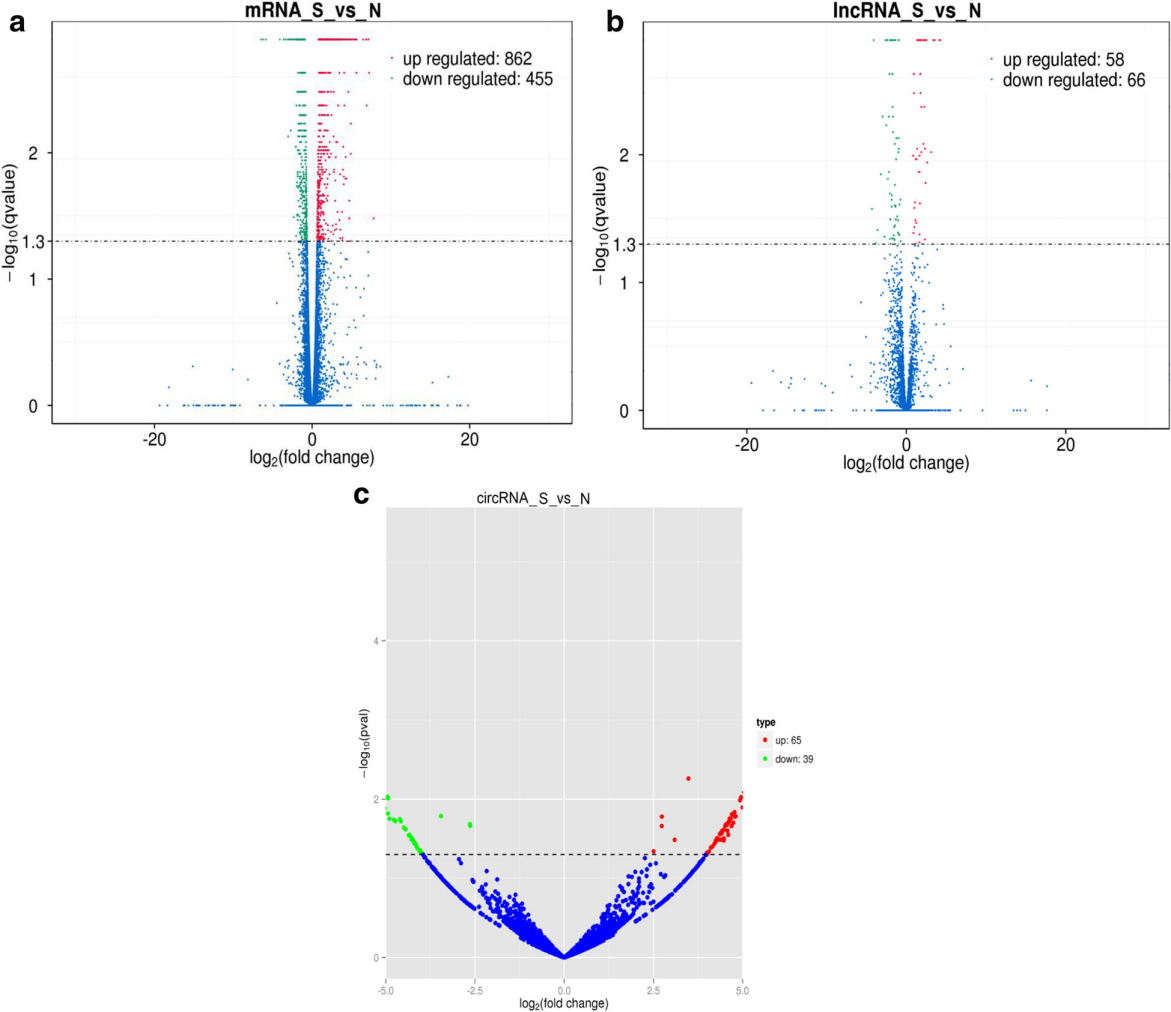
Differentially expressed mRNAs (**a**), lncRNAs (**b**) and circRNAs (**c**) in the two groups. Volcano plot of the *p-*values as a function of fold-change for mRNAs, lncRNAs and circRNAs indicate the dysregulated genes in the three normal and three infected tissues. Blue dots represent RNAs not significantly differentially expressed (*p-*value > 0.05) and the other dots represent RNAs differentially expressed (*p-*value < 0.05). Up-regulated RNAs were presented as red dots and down-regulated RNAs were presented as green dots

The number of overlap mRNAs, lncRNAs and circRNAs in the experimental group compared with the control group is displayed in the Venn diagram, respectively (Fig. 2a–c). A total of 1317 mRNAs and 124 lncRNAs (Additional file 2) were found to be differentially expressed with the *q*-value < 0.05 and FDR < 0.05. Among them, 862 mRNAs were upregulated and 455 were downregulated in three infectious tracheal tissues compared with the controls (Fig. 3a). Meanwhile, 58 and 66 lncRNAs were upregulated and downregulated, respectively (Fig. 3b). According to the criteria of fold change > 2.0, 656 mRNAs were up-regulated and 292 were down-regulated. Similarly, 53 and 57 lncRNAs were up-regulated and down-regulated. For circRNAs, 104 remarkably differentially expressed genes (fold change > 2.0, *p*-value < 0.05 and FDR < 0.05) between two groups were identified (Additional file 2). Among them, 65 circRNAs were up-regulated and 39 were down-regulated (Fig. 3c).

**Fig. 4:**
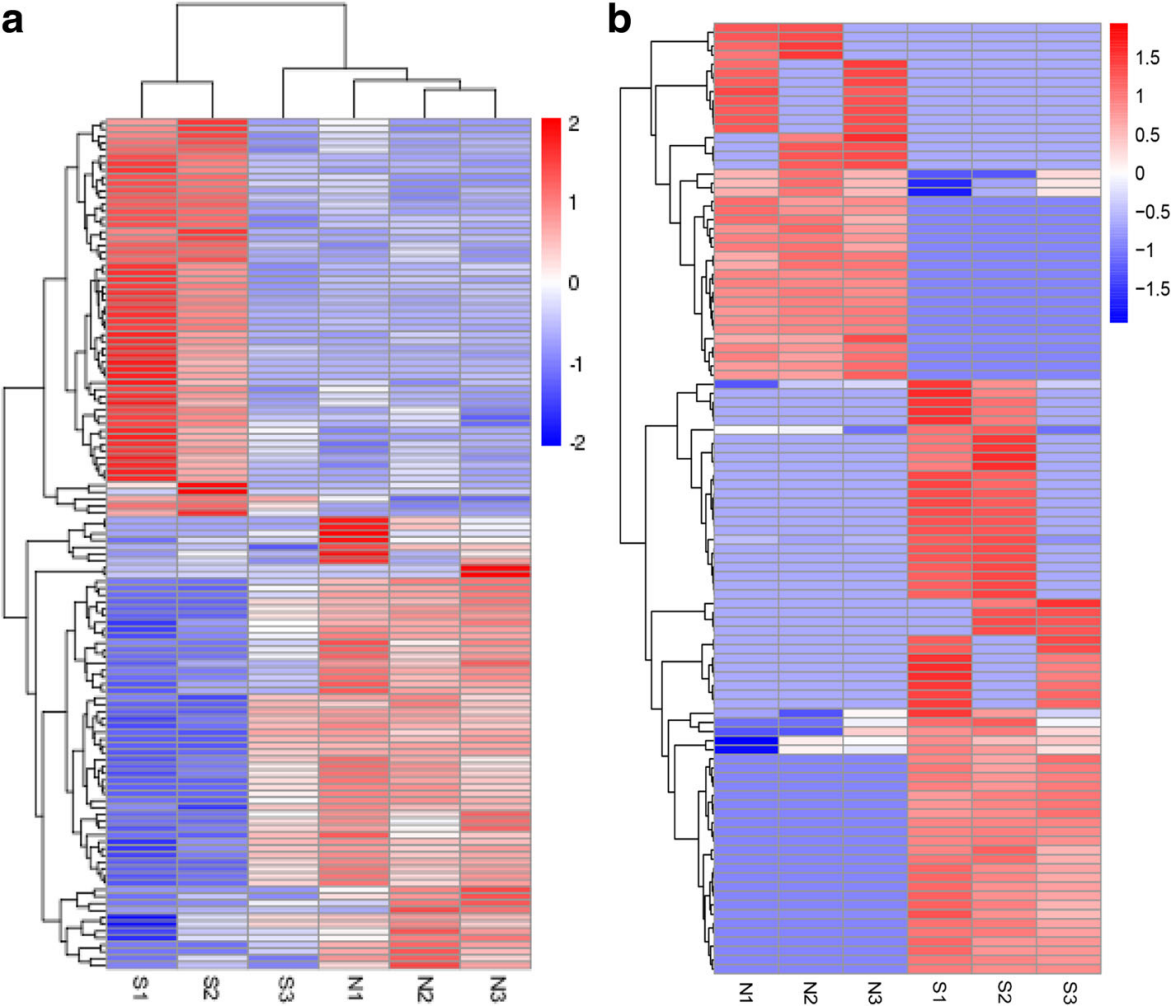
Heatmap of expression profiles for the lncRNAs (**a**) and circRNAs (**b**). Red through blue color indicates high to low expression level. Each column represents one sample, and each row indicates a transcript

In order to further understand the similarities among tracheas of chickens at the transcriptomic level, the data of all the differentially expressed genes were used for cluster analysis. The heat map clearly showed that the experimental group and the control group were separately clustered together (Fig. 4). The expression patterns of lncRNAs and circRNAs between the experimental group and the control group were distinguishable. These data suggested that the expressions of lncRNAs and circRNAs in chicken tissues infected with *C. baileyi* were significantly different from those in non-infected chicken tissues.

### Comparison of mRNAs and lncRNAs features

**Fig. 5:**
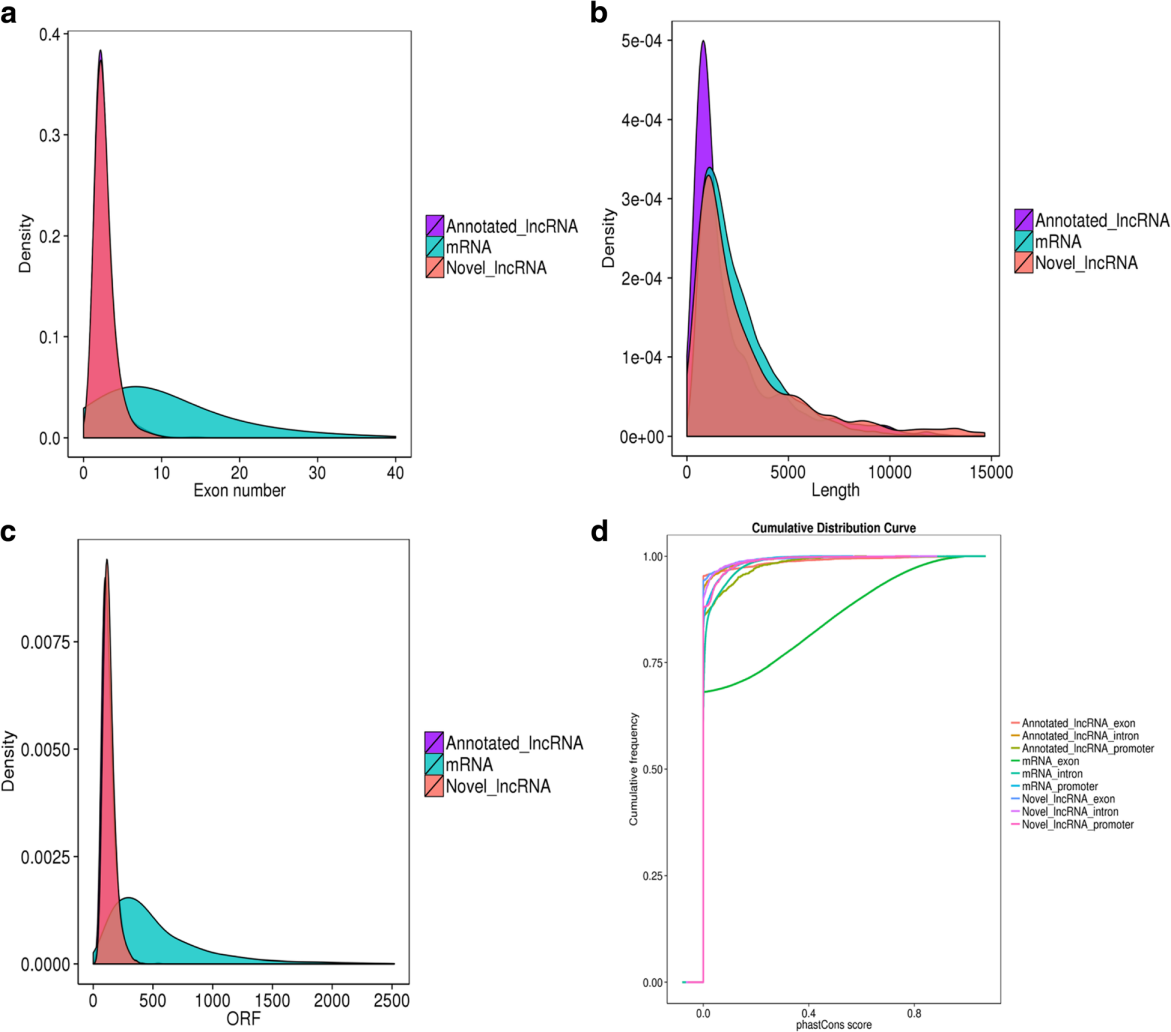
Comparison of genomic architecture between mRNAs and lncRNAs. **a** The number of exons in the mRNAs and lncRNAs. In present study, single-exon lncRNAs were filtered out from the chicken genome due to the limitations of the algorithm. **b** Distribution of transcript lengths in the mRNAs and lncRNAs. The horizontal axis of indicates the length of transcripts, and the vertical axis represents density. **c** The number of open reading frames (ORFs) in the mRNAs and lncRNAs. **d** Conservation of the sequence in mRNAs and lncRNAs were evaluated using phastCons

In this study, a total of 14,698 mRNAs, 1616 annotated lncRNAs and 1376 novel lncRNAs were identified from the tracheal tissues of chickens after infection with *C. baileyi*. To examine the differences among these three kinds of transcripts, comparative analysis of gene structure and sequence conservation was conducted. Our results showed that most lncRNAs contained two or three exons, which was significantly less than that of mRNAs (Fig. 5a). However, a slightly discrepancy was observed in the distribution of transcript length between mRNAs and lncRNAs (Fig. 5b). Compared with mRNAs, a relatively shorter open reading frame (ORF) was one of the main features of most lncRNAs (Fig. 5c). In addition, the majority of lncRNAs were less conserved which was different with mRNAs, although the difference is not statistically significant (Fig. 5d).

### Validation of dysregulated mRNAs, lncRNAs and circRNAs

**Fig. 6:**
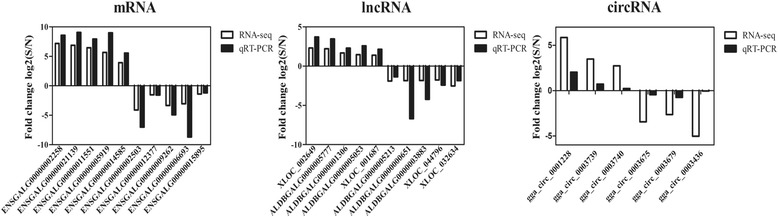
Comparison of results of RNA sequencing and qRT-PCR. The blank column represents the fold change values obtained from RNA sequencing and the black column displays the fold change values of qRT-PCR. The figures represent the comparison results of mRNAs, lncRNAs and circRNAs from left to right. *GAPDH* was used as the internal control gene and three biological replicates were used. The expression trends of the results of RNA-seq were consistent with qRT-PCR

Ten mRNAs, ten lncRNAs and six circRNAs were randomly selected from the dysregulated genes to be verified by qRT-PCR. It was demonstrated that the expression levels from qRT-PCR data were consistent with the results of RNA sequencing (RNA-seq) (Fig. 6) thus verified the facticity of RNA-seq analysis. Consequently, efficient evidence was provided by this finding that these mRNAs, lncRNAs and circRNAs could be used to investigate the pathogenesis of *C. baileyi* infection in the future.

### Co-expression analysis and target prediction

**Fig. 7:**
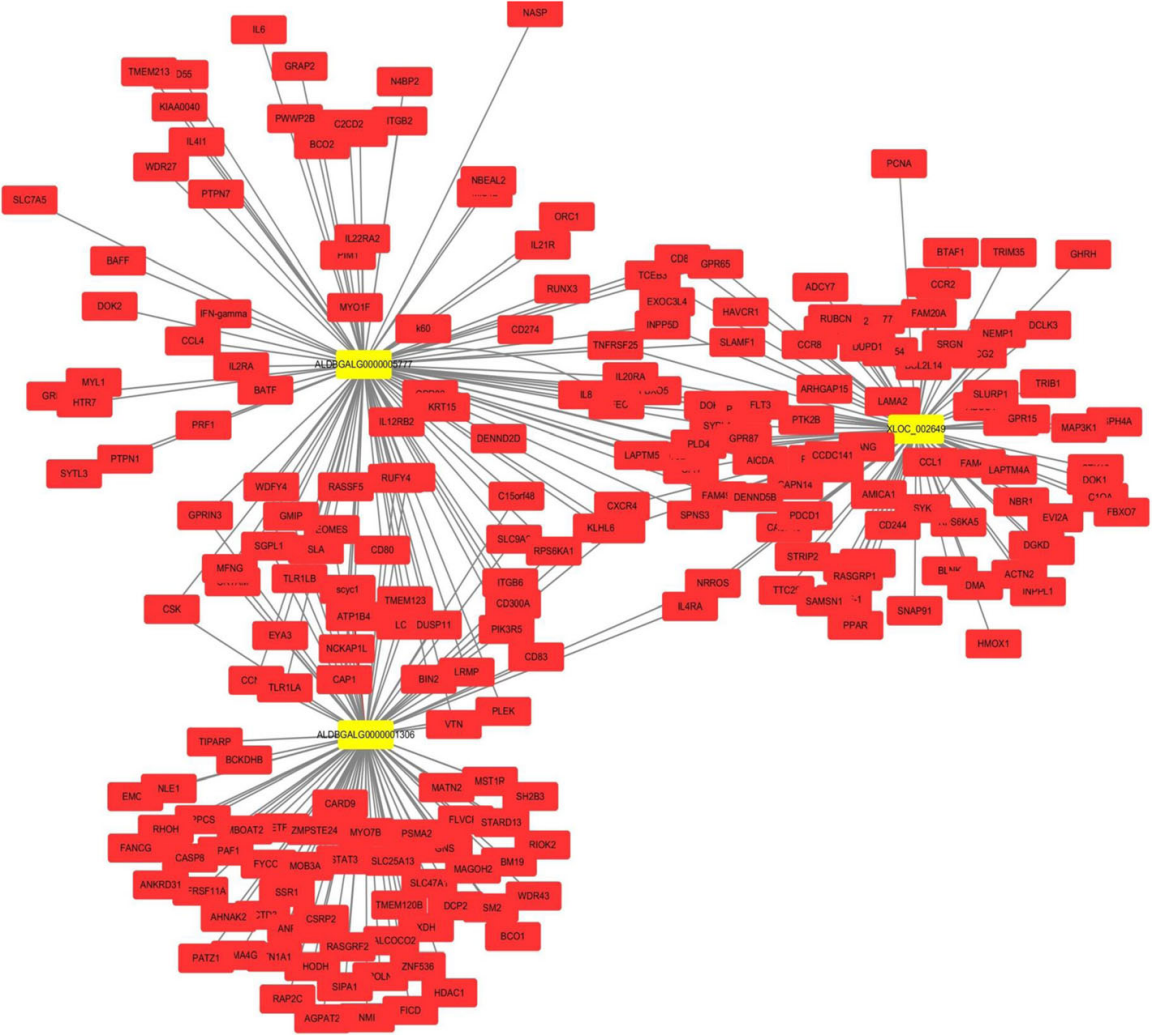
Co-expression network of the representative lncRNAs and their partial target mRNAs. The relationships of mRNA-lncRNA were re-constructed based on the expression correlation coefficients (Pearson correlation > 0.95 or < − 0.95) using the cytoscape software (v3.6.0). Different colors were used to show different genes, with yellow for lncRNAs and red for mRNAs

Generally, the functions of both lncRNAs and circRNAs were performed by inter-acting with their targets [27, 28]. In our study, the potential *cis-* and *trans-*targets were predicted (Additional file 3)*.* The mRNAs 100 kb upstream and downstream of the lncRNAs were searched for *cis* analysis, with 2308 lncRNAs that corresponded to 7679 protein-coding genes. The *trans* analysis of lncRNAs was performed by constructing co-expression networks (Fig. 7) of dysregulated mRNAs and lncRNAs based on expression correlation coefficient (Pearson correlation > 0.95 or < − 0.95) (Additional file 3). A total of 14,418 mRNAs and 2570 lncRNAs containing 634,780 relationships were detected. More than one mRNAs were predicted to be regulated by one lncRNA, and one mRNA corresponded to several lncRNAs (Fig. 7).

Previous studies have indicated that miRNA sponge was the common function of circRNAs [43, 44]. However, we have not examined the expression profile of miRNA in chicken tracheas during *C. baileyi* infection. Therefore, we just predicted the potential miRNA targets based on sequence complementarity in the present study, and the possible interaction relationships between 17,607 circRNAs and the 978 miRNAs in the chicken genome were observed (Additional file 4).

To further predict the function of mRNAs, lncRNAs and circRNAs from tracheal tissue of chickens, the GO (http://www.geneontology.org/) (Additional file 5) and KEGG (http://www.genome.jp/kegg/) pathway analyses (Additional file 6) were performed to analyze the dysregulated mRNAs and the target genes of differentially expressed lncRNAs and the source genes of circRNAs between the two groups.

**Fig. 8:**
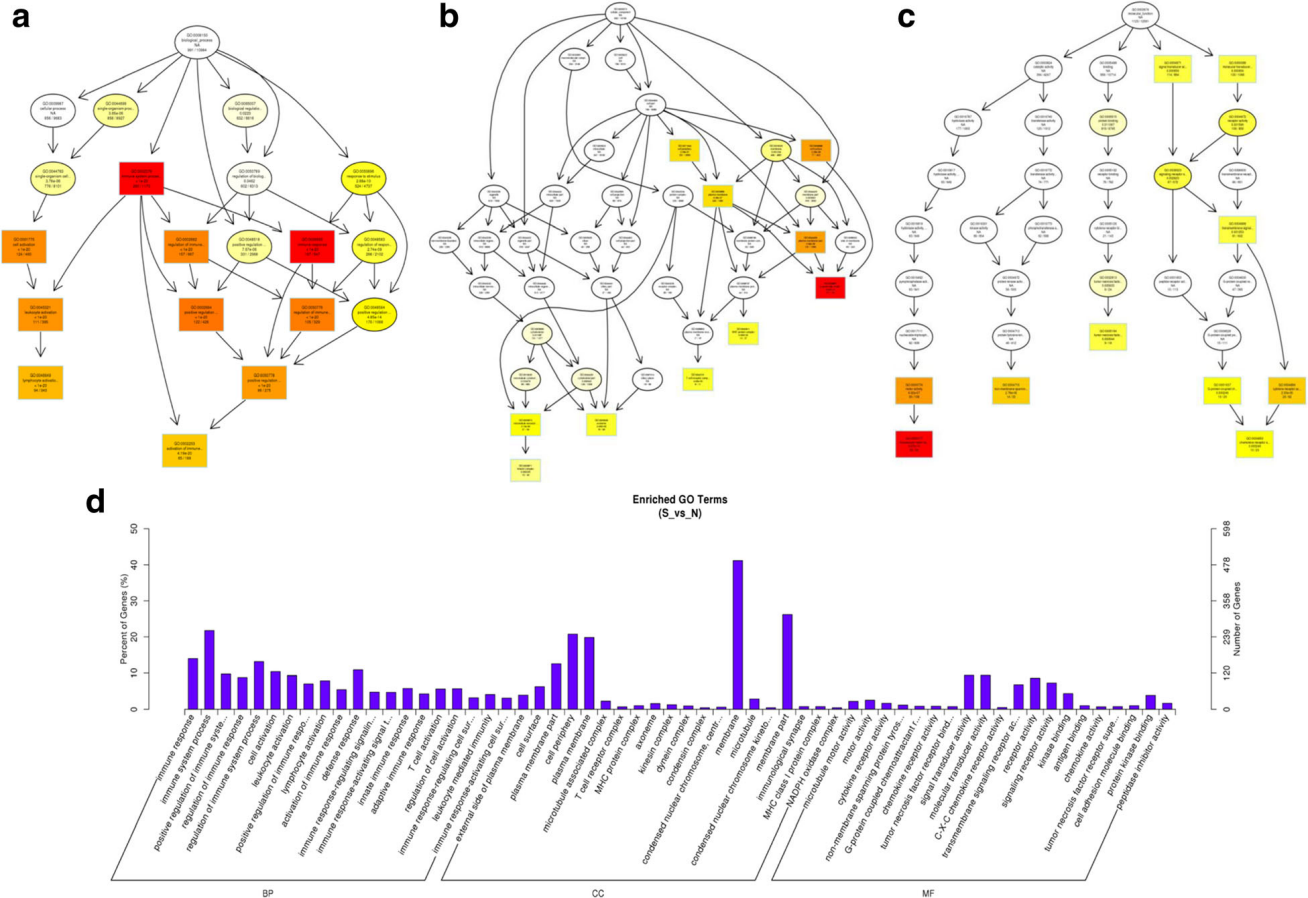
GO analysis of mRNAs in trachea tissue of chickens. The significant enriched biological process, cellular component and molecular function with changed mRNAs in trachea tissue of chicken were displayed. Directed Acyclic Graph (DAG) is the graphical display of GO enrichment results with candidate targeted genes (**a**–**c**). The number of genes in GO term were showed in histograph (**d**)

**Fig. 9:**
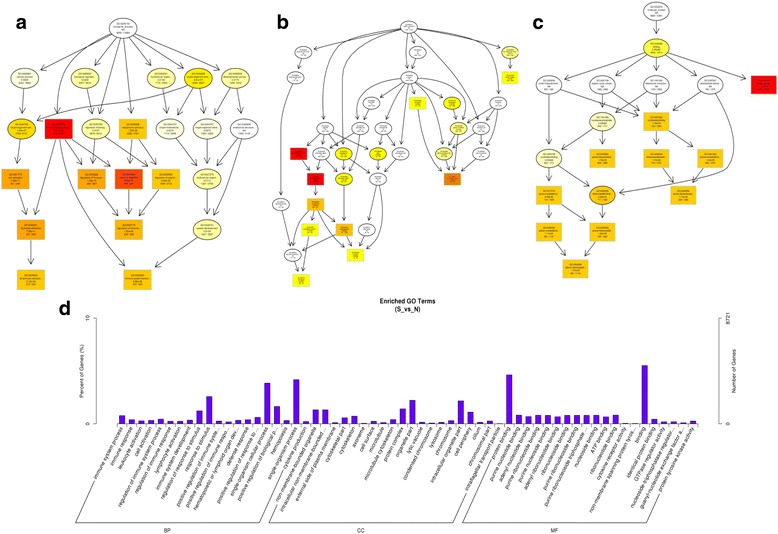
GO analysis of lncRNAs in trachea tissue of chickens. The significant enriched biological process, cellular component and molecular function with changed lncRNAs in trachea tissue of chicken were shown. Directed Acyclic Graph (DAG) is the graphical display of GO enrichment results with candidate targeted genes. The branch represents the relationship of inclusion, which defines the scope from increasingly small from top to bottom. The color depth represents the enrichment degree (**a**–**c**). The number of genes in GO term were showed in histograph (**d**)

In the present study, the top 10 results of GO enrichment analysis were selected as the master node of directed acyclic graph (DAG). The DAG of biological process (BP), cellular component (CC) and molecular function (MF) of differentially expressed mRNAs and lncRNAs are shown in Figs. 8a–c and 9a–c, respectively. Based on the GO analysis of differentially expressed mRNAs, the most significantly enriched BP were immune response, immune system process and positive regulation of immune system process, and external side of plasma membrane, cell surface and plasma membrane part were the most enriched CC. Furthermore, microtubule motor activity, motor activity and cytokine receptor activity were identified to be the most significantly enriched MF (Fig. 8d). On the basis of GO analysis of the target genes of differentially expressed lncRNAs, the most significantly enriched BP were immune system process, immune response and leukocyte activation, and the non-membrane-bounded organelle, intracellular non-membrane-bounded organelle and external side of plasma membrane were the most remarkably enriched CC. In addition, protein binding, purine nucleotide binding and adenyl nucleotide binding were the most significantly enriched MF (Fig. 9d). The most prominent category of gene function will be the focus of future research. Since the numbers of up-regulated and down-regulated circRNAs were small, there were no significant GO terms enriched in the two groups.

**Fig. 10:**
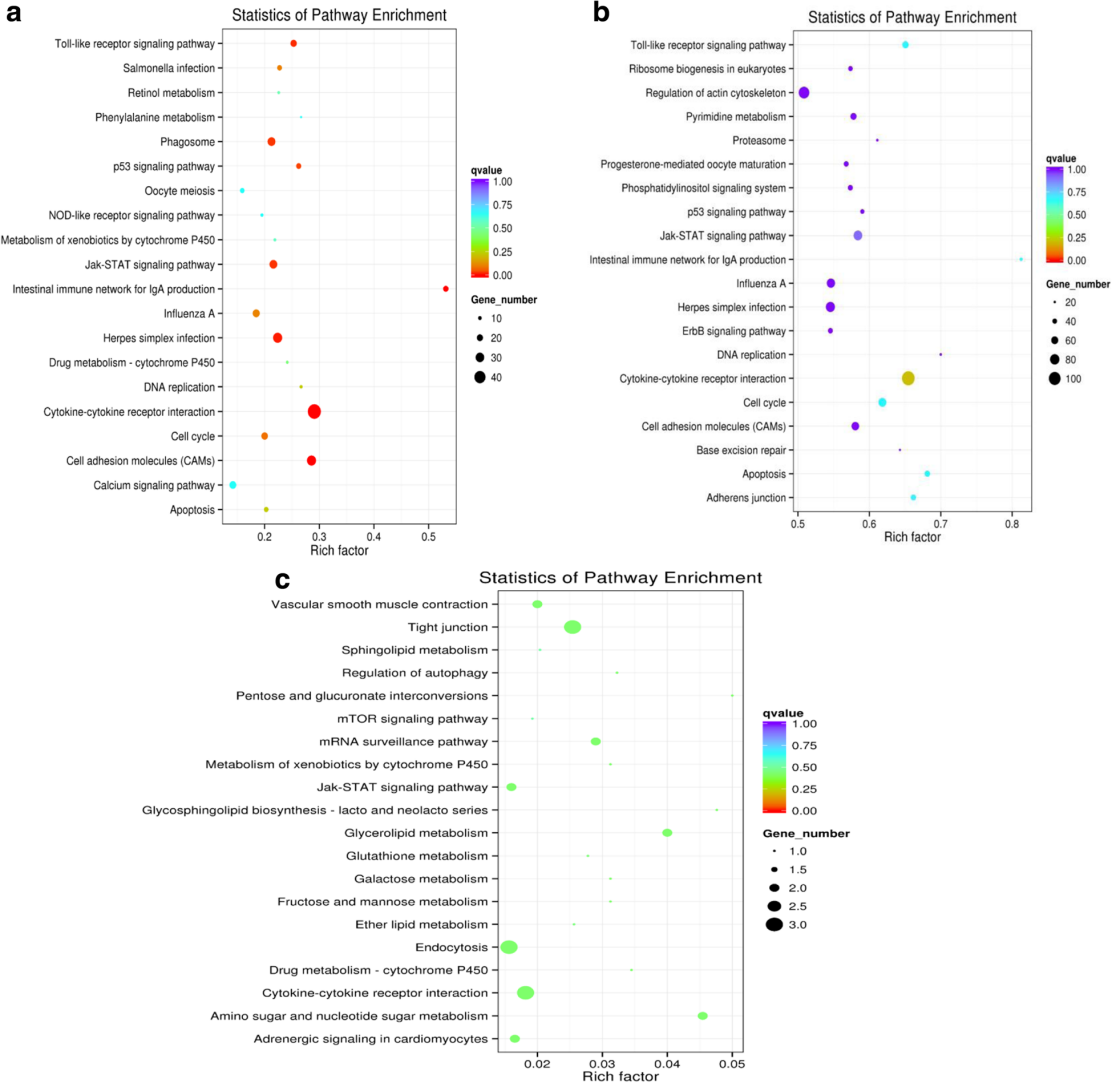
mRNAs (**a**), lncRNAs (**b**) and circRNAs (**c**) enriched KEGG pathway scatterplot showing statistics of pathway enrichment in the tracheal tissue of chickens between the two groups. The vertical axis represents the pathway name and the horizontal axis represents the rich factor. The size of the dot represents the number of differentially expressed genes in the pathway and the color of the point corresponds to the different *q*-value range

As a major public database of pathways, KEGG has been used to determine the significantly enrichment pathways for candidate target genes compared with the entire genome background [51, 52]. The top 20 pathways associated with mRNAs, lncRNAs and circRNAs were showed in an enriched scatter diagram, respectively. For mRNAs, the cytokine-cytokine receptor interaction, intestinal immune network for IgA production and cell adhesion molecules (CAMs) were identified as the top enriched KEGG pathways (Fig. 10a). KEGG pathway enrichment analysis for significantly differentially expressed lncRNAs revealed pathways for intestinal immune network for IgA production, cytokine-cytokine receptor interaction and cell cycle (Fig. 10b). Whereas, the significantly enriched pathways were amino sugar and nucleotide sugar metabolism, tight junction and glycerolipid metabolism for circRNAs (Fig. 10c).

## Discussion

*C. baileyi* has been considered to be the most common avian *Cryptosporidium* species worldwide with the broadest host range [7, 53]. It can reside in the epithelial cells of respiratory tract, causing clinical respiratory disorders [4, 10, 11, 54] in birds (eg. chickens, turkeys and ducks). Considering the distinct morphological and biological features and large oocyst production, *C. baileyi* has been suggested as a model for the characterization of cryptosporidia [55, 56]. Moreover, *C. baileyi* is able to establish its infection in the mucosal epithelium of a wide variety of organs (eg. respiratory tract, bursa of Fabricius and cloaca) [4, 53], and the trachea is the most common predilection site to present the inflammation and clinical signs. Therefore, the trachea from the experimental and control groups were selected to study the differentially expressed profiles of mRNAs, lncRNAs and circRNAs. The results of our study indicated that mRNAs and ncRNAs may play a significant role in the infection process of *C. baileyi*, representing a potential therapeutic target for *C. baileyi* infection as revealed by the sequencing analysis.

The chicken genome has been sequenced and assembled using the whole-genome mapping technology in 2004 [57, 58], with high-quality genome assembly and annotation. The novel mRNAs, lncRNAs and circRNAs obtained in our study greatly improved the annotation of the chicken genome. A total of 14,698 mRNAs were obtained from the tracheal tissue of chickens infected with *C. baileyi* and 1317 were significantly dysregulated. The pathways of cytokine-cytokine receptor interaction, CAMs and toll-like receptor signaling were significantly enriched by GO and KEGG enrichment analyses of these differentially expressed mRNAs, and previous studies also indicated that the infection of zoonotic *C. parvum* was closely associated with these pathways in the epithelial cells [59–61], indicating that these dysregulated genes may be involved in the interactions between the host and *C. baileyi*.

The potential functions of lncRNAs were commonly predicted by their target genes. In our study, the relationships between lncRNAs and their target genes co-located (within 100 kb) and co-expressed (the Pearson’s correlation coefficients > 0.95 or < − 0.95) were analyzed (Additional file 3, Fig. 7). Analyzing potential target genes of dysregulated lncRNAs revealed their important roles in regulation of the interaction between chickens and *C. baileyi*. For example, one lncRNA, ALDBGALG0000001306, was significantly upregulated between the infected and non-infected chickens, and its potential targets IL-6, IL-12 and IL-17 have been identified as important cytokines against *Cryptosporidium* infection [53, 62–64], suggesting that this lncRNA would be a regulator of immune response against *C. baileyi* infection in chickens.

Additionally, circRNAs, a newly discovered class of ncRNAs, may act as miRNA sponges to regulate the activity of target genes and participates in the regulation of gene transcription and protein production [65, 66] to affect prognosis of diseases, especially tumors [47, 49]. Therefore, circRNAs were tested in our study using RNA sequencing and only 104 circRNAs genes (65 were upregulated and 39 were downregulated) were demonstrated to be differentially expressed in response to the infection of *C. baileyi*. Compared with mRNAs and lncRNAs, the expression levels of circRNAs were generally lower (Fig. 6) and no significant GO terms were enriched in our study. The reasons for these differences may be due to the drawbacks of RNA sequencing protocol in our study or the expression of circRNA might be closely related to immune-specific organs and tissues (eg. brain, stem cells, testis and discs) predicted in previous studies [67–69]. The source genes of these circRNAs were mainly enriched in the metabolic pathway of amino sugar and nucleotide sugar metabolism, tight junction and glycerolipid metabolism. Some miRNAs (eg. let-7d, let-7f and let-7i) have been proven to regulate the expression of toll-like receptor four signal in respond to *C. parvum* infection [25, 26, 70, 71], and their interaction circRNAs (Additional file 4) were also found to be differentially expressed, suggesting that these circRNAs may implicate in the pathogenesis and progression of *C. baileyi* infection.

## Conclusions

Differentially expressed mRNAs, lncRNAs and circRNAs were screened from chickens after *C. baileyi* infection. A total of 1317 mRNAs, 124 lncRNAs and 104 circRNAs were differentially expressed, and these RNAs could regulate expression of their related genes and play a key role in the pathogenesis of *C. baileyi* infection. The relevant signaling pathway of these predicted mRNAs and ncRNAs would be the focus of future study to fully reveal the pathogenesis of *C. baileyi* infection. The current work also provides new insight into the pathways and mechanisms that mediate the host immune response to *C. baileyi* (albeit in birds only).

## Methods

### *C. baileyi* infection model

A total of six newly hatched chickens were purchased from the Giant Long Company (Shaanxi, China), and reared in a pathogen-free laboratory and given constant light and adequate food and water during the entire experimental period. Chickens were divided into two groups randomly with three chickens per group and each chicken was kept in a separate cage. After normal feeding for 3 days, each chicken in the experimental group (S group) was orally infected with 1 × 10^6^ *C. baileyi* oocysts, while the control group (N group) was given the same volume of PBS. Faecal samples were collected daily post infection (pi). The hemacytometer was used to record the numbers of oocyst per gram (OPG) of faeces. The successful infection was confirmed by the oocyst excretion (Additional file 7) and parasites in histological observations by haemotoxylin & eosinstaining and by electron microscopy (Additional file 8).

### Sample collection and preparation

Since *C. baileyi* mainly parasitizes in the respiratory tract, causing a series of respiratory diseases, at the first peak of oocyst excretion (10 dpi), the tracheas of three chickens in both experimental and control groups were collected and sent to the company (Novogene, Beijing) for sequencing. Chickens were firstly kept into inverted beakers with infiltrated ether ball for several minutes. When they presented symptoms of slow breathing, absent corneal reflexes and skin sensation, these animals were then anesthetized. The trachea from each chicken was rapidly sampled and washed with nuclease-free PBS. All experiments were permitted by ethics committee of Northwest A&F University and conducted in the fume hood. All the instruments and reagents were treated with DEPC in advance and without RNase.

### RNA isolation, library preparation and sequencing

Total RNA was extracted from each trachea sample using TRIzol reagent (Invitrogen, Carlsbad, CA, USA) according to the manufacturer’s instructions. The concentration and purity of RNA were examined using the Qubit® RNA Assay Kit in Qubit® 2.0 Flurometer (Life Technologies, CA, USA) and the NanoPhotometer® spectrophotometer (IMPLEN, CA, USA), respectively. The RNA integrity was measured using the RNA Nano 6000 Assay Kit of the Bioanalyzer 2100 system (Agilent Technologies, CA, USA). A total amount of 3 μg RNA per sample was then used for RNA sequencing. Firstly, the ribosomal RNA (rRNA) was removed by Epicentre Ribo-zero™ rRNA Removal Kit (Epicentre, USA). Subsequently, the RNAs were subjected to generate sequencing libraries by NEBNext® Ultra™ Directional RNA Library Prep Kit (NEB, USA). After that, the first and second strand of the complementary DNA (cDNA) were synthesized and the AMPure XP system (Beckman Coulter, Beverly, USA) was used to purify the library fragments for firstly selecting cDNA fragments with a length of 150–200 bp. PCR was then performed and the Agilent Bioanalyzer 2100 system was used to evaluate the quality of the library. HiSeq PE Cluster Kit v4 cBot (Illumina) was used to perform the clustering of index-coded samples according to the manufacturer’s instructions. The libraries were sequenced on an Illumina Hiseq 4000 platform and 150 bp paired-end reads were produced after cluster generation. For circRNA sequencing, the RNase R (Epicentre, USA) was additionally used to deal with the rRNA-depleted RNAs and remove the linear RNAs before sequencing library generation.

### Quality control

The raw reads were processed firstly by the in-house perl scripts. To obtain clean reads, low quality reads, containing adapter and ploy-N reads were removed from raw data in this step. Meanwhile, the GC content, Q20 and Q30 of the clean data were also calculated. The clean data with high quality was used for further analysis.

### Mapping to the reference genome

The reference genome (ftp://ftp.ensembl.org/pub/release-83/fasta/gallus_gallus/dna/) and gene model annotation files (ftp://ftp.ensembl.org/pub/release-83/gtf/gallus_gallus/) were downloaded from website directly. Bowtie (v2.0.6) [72] was used to build index of the reference genome, and TopHat (v2.0.9) [73] was used to align paired-end clean reads to the reference genome. In addition, the software find_circ [74] was used to extend the anchor sequences and the back-spliced reads containing at least two supporting reads were considered to be circRNAs.

### Transcriptome assembly of lncRNAs

The softwares Scripture beta2 [75] and Cufflinks (v2.1.1) [76, 77] were used to assemble mapped reads of each sample. These two methods determined exons connectivity by using spliced reads in different ways. With setting other parameters as default, Scripture and Cufflinks were run with ‘min-frags-per-transfrag = 0’ and ‘–library-type’, respectively.

### LncRNAs coding potential and conservation analysis

Four analytic tools, namely coding-non-coding-index (CNCI) [78], coding potential calculator (CPC) [79], phylogenetic codon substitution frequency (PhyloCSF) [80] and Pfam-scan [81], were used to effectively distinguish protein-coding and non-coding sequences. Ultimately, the transcripts predicted with coding potential by any of the four tools were filtered out, and those without coding potential would to be our candidate set of lncRNAs.

The software phast (v1.3) [82] was usually used for phylogenetic analysis to evaluate the sequence conservation of transcripts. Then, we used the program phyloFit to calculate phylogenetic models of the conserved and non-conserved regions among species. PhastCons was used to compute the conservation scores of coding genes and lncRNAs.

### Target gene prediction and differential expression analysis

Both *cis* and *trans* roles of target genes for lncRNA were predicted. Here, we searched coding genes within 100 kb upstream and downstream of each lncRNA and then analyzed their function. Fragments per kilobase for a million reads (FPKMs) of both lncRNAs and coding genes in each sample [77] were calculated by the Cuffdiff (v2.1.1). Unlike the former, psRobot [83] was used to predict the miRNA binding sites of circRNAs. Besides, the transcript per million (TPM) was used to normalize the expression level of circRNAs according to the criteria described by Zhou et al. [84]. Between two groups of chickens, transcripts or genes with *p*-value adjusted by the Benjamini & Hochberg method (*q*-value) < 0.05 were considered significant.

### GO and KEGG enrichment analysis

To explore the function of mRNAs, lncRNAs and circRNAs, the Gene Ontology (GO) and Kyoko Encyclopedia of Genes and Genomes (KEGG) enrichment analysis were conducted. The GOseq (v1.18.0) was used to perform the GO enrichment analysis of differentially expressed genes or target genes of lncRNAs or source genes of differentially expressed circRNAs, in which gene length bias was corrected. All three Go categories, namely cellular component, biological process, and molecular function, were included, and GO terms with the *q*-value < 0.05 was considered significantly enriched. KOBAS [51] software was used to examine the statistical enrichment analysis of differential expression genes or lncRNA target genes or source genes of differentially expressed circRNAs in KEGG pathways. The gene sets were firstly mapped to database genes, and then compared with reference chicken genome (ftp://ftp.ensembl.org/pub/release-83/fasta/gallus_gallus/dna/) to scan enriched pathways, diseases and functions. The enriched information was then evaluated by the statistical test and correction. The EASE score was calculated to test the relevance, and *p*-value < 0.05 was considered significantly enriched by differentially expressed genes.

### Validation by quantitative real-time polymerase chain reaction (qRT-PCR)

Three trachea RNA samples from both infected and non-infected chickens were analyzed by qRT-PCR. Total cDNA of each sample was synthesized using two-step reverse transcriptase Kit (Vazyme Biotech Co., Ltd., Nanjing, China) according to the manufacturer’s instructions. qRT-PCR were performed using LightCycler 480II Real-Time PCR System (Roche, Indianapolis, Indiana) and UltraSYBR Mixture (Qiagen, Shanghai, China). Each reaction (in 10 μL) contained 5 μL 2 × QuantiFast® SYBR® Green PCR Master Mix, 0.4 μL primers (5 μM) (Additional file 9), and 1 μL cDNA. The protocol included an initial single cycle denaturing at 95 °C for 10 min, followed by 40 cycles of denaturing at 95 °C for 10 s and annealing at 60 °C or 55 °C (Additional file 9) for 30 s. All amplifications were followed by dissociation curve analysis of the amplified products. Specific primers were designed using the Primer Premier (v5.0), and specificities were confirmed with BLAST. The gene expression levels were normalized with the reference gene *GAPDH* by using 2^−ΔΔCt^ value methods. The data were presented as the means ± SEM.

### Statistical analysis

The Pearson’s correlation coefficients were used to calculate expression correlation between lncRNAs and mRNAs (*r* > 0.95 or *r* < − 0.95). The statistical difference in gene expression of qRT-PCR results was analyzed by Student’s t-test and false discovery rate (FDR) was also calculated to correct the *p*-value. It was considered to be statistically significant when *p*-value < 0.05 or *q*-value < 0.05.

## Additional files

Additional file 1: The information of all novel lncRNAs identified in this study. (XLSX 106 kb)

Additional file 2: All the differentially expressed mRNAs, lncRNAs and circRNAs in this study. (XLSX 150 kb)

Additional file 3: LncRNA-protein coding gene pairs with co-location and co-expression relationships. (XLSX 13272 kb)

Additional file 4: The results of circRNA-binding miRNAs analysis. (XLSX 33940 kb)

Additional file 5: GO enrichment analysis of mRNAs and lncRNAs. (XLS 18518 kb)

Additional file 6: KEGG pathway analysis of mRNAs, lncRNAs and circRNAs. (XLS 255 kb)

Additional file 7: The oocyst excretion of chickens infected with *C. baileyi*. The horizontal axis represents the day post infection (dpi) with *C. baileyi* oocysts of chickens and the vertical axis shows the number of OPG. The oocysts were detected in faeces of infected chickens from 5 dpi, and the peaks of oocyst excretion were observed at 10 and 16 dpi. (TIF 6095 kb)

Additional file 8: The parasites in histological observations by haemotoxylin & eosin staining (A and B) and by electron microscopy (C and D). No parasite was detected in the tracheas of the control chickens with haemotoxylin & eosinstaining (A, × 400) and electron microscopy (C, × 2000), but there were a lot of parasites in the tracheas of the experimental chickens (B and D). (TIF 4763 kb)

Additional file 9: Primers used in qRT-PCR analysis. (XLSX 10 kb)

## Abbreviations

BP: Biological process
*C. baileyi*: *Cryptosporidium baileyi*
*C. parvum*: *Cryptosporidium parvum*
CAMs: Cell adhesion molecules
CC: Cellular component
cDNA: Complementary DNA
circRNAs: Circular RNAs
CNCI: Coding-non-coding-index
CPC: Coding potential calculator
DAG: Directed acyclic graph
FDR: False discovery rate
FPKMs: Fragments per kilobase for a million reads
GO: Gene ontology
KEGG: Kyoto Encyclopedia of Genes and Genomes
lincRNAs: Long intergenic non-coding RNAs
lncRNAs: Long non-coding RNAs
MF: Molecular function
miRNA: microRNA
ncRNA: Non-coding RNA
OPG: Oocyst per gram
ORF: Open reading frame
PhyloCSF: Phylogenetic codon substitution frequency
pi: Post infection
qRT-PCR: Quantitative real-time polymerase chain reaction
RNA-seq: RNA sequencing
rRNA: Ribosomal RNA
SEM: Standard error of mean
TPM: Transcript per million
