# Supplementary figures and images for: Genome-wide analysis of differentially expressed profiles of mRNAs, lncRNAs and circRNAs during Cryptosporidium baileyi infection
Source: BMC Genomics. 2018 May 10;19:356. doi: 10.1186/s12864-018-4754-2 (PMC5946474; doi:10.1186/s12864-018-4754-2)

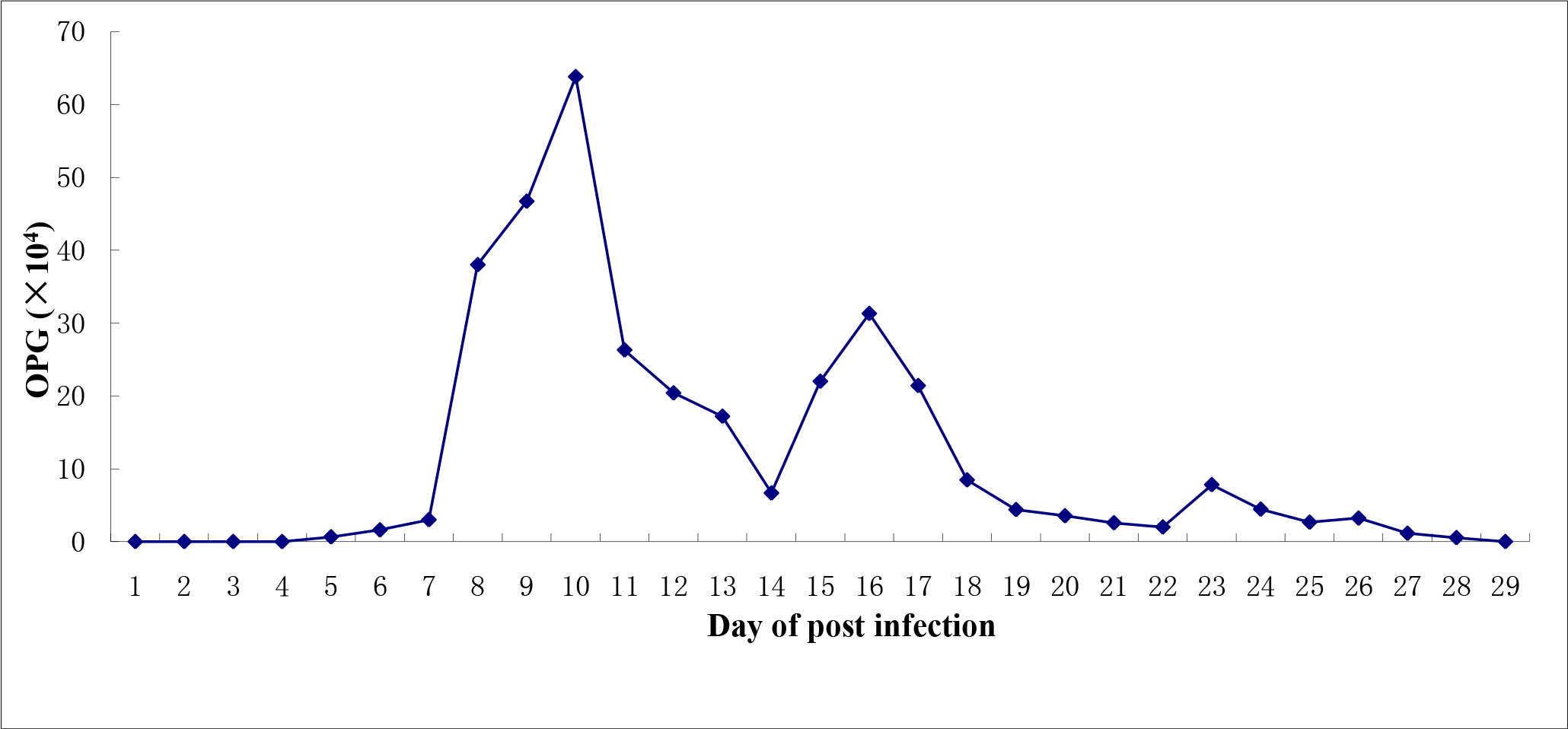

Supplement: Supplementary file 7 — The oocyst excretion of chickens infected with C. baileyi. The horizontal axis represents the day post infection (dpi) with C. baileyi oocysts of chickens and the vertical axis shows the number of OPG. The oocysts were detected in faeces of infected chickens from 5 dpi, and the peaks of oocyst excretion were observed at 10 and 16 dpi. (TIF 6095 kb) [file 12864_2018_4754_MOESM7_ESM.tif]

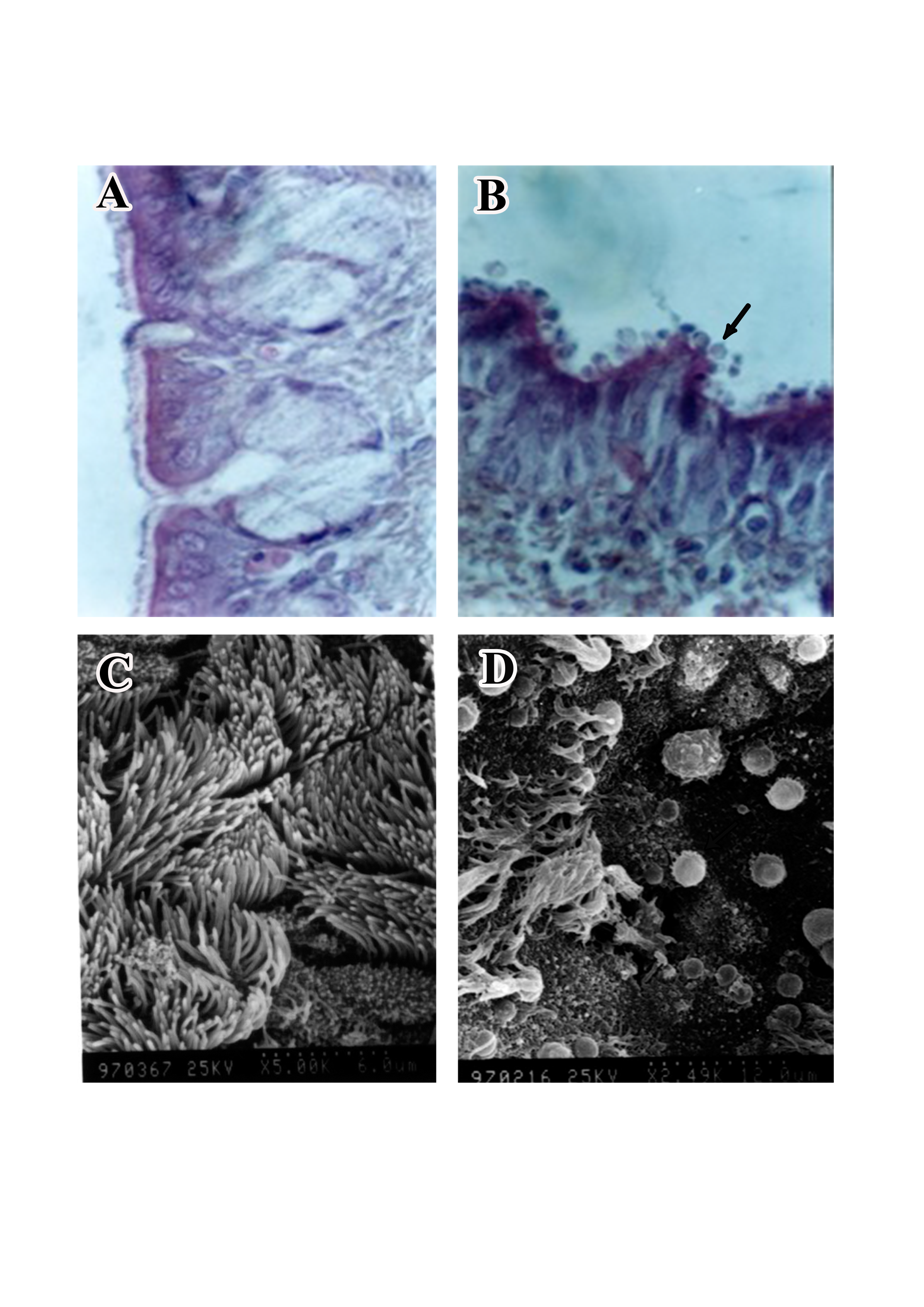

Supplement: Supplementary file 8 — The parasites in histological observations by haemotoxylin & eosin staining (A and B) and by electron microscopy (C and D). No parasite was detected in the tracheas of the control chickens with haemotoxylin & eosinstaining (A, × 400) and electron microscopy (C, × 2000), but there were a lot of parasites in the tracheas of the experimental chickens (B and D). (TIF 4763 kb) [file 12864_2018_4754_MOESM8_ESM.tif]
